# Anemia revealing a collagenous gastritis in a young Tunisian man

**DOI:** 10.11604/pamj.2018.30.231.12981

**Published:** 2018-07-26

**Authors:** Imen Akkari, Karim Skandrani, Atef Ben Abdelkader, Soumaya Mrabet, Elhem Ben Jazia

**Affiliations:** 1 Gastroenterology Department, Farhat Hached Hospital, Sousse, Tunisia; 2 Anatomopathology Department, Farhat Hached Hospital, Sousse, Tunisia

**Keywords:** Gastritis, collagen diseases, endoscopy, anemia, iron deficiency

## Abstract

Collagenous gastritis is a rare entity, characterized by the deposition of a subepithelial collagenous band with an inflammatory infiltrate in the mucosa. We report the first Tunisian case revealed by severe anemia. Lesions were limited to the stomach and remained unchanged on 3 series biopsies during a 24 month follow up despite treatment with corticosteroids. The cause of the disease remains unknown; our findings suggest that lesions of collagenous gastritis may result from a local immune process.

## Introduction

Collagenous gastritis (CG) is a very rare and benign disease of unknown origin, characterized by thickening of the subepithelial collagenous band (more than 10 μm in thickness) in the gastric mucosa associated with a mixed inflammatory infiltrate within the lamina propria [1]. To our knowledge, there have been approximately 60 cases reported in the English literature [2]. Since it was initially described by Colletti and al in 1989 [3]. Authors divide CG into two subtypes. The first one occurs in children and young adults presenting with severe anemia, a nodular gastric pattern on endoscopy. The second subtype occurs mainly in adults characterized by a watery diarrhea [4,5]. Because of its unclear etiopathogenesis, there is no clear established therapeutic strategy, and both corticoids and proton pump inhibitors (PPIs) are presently the drugs of choice [6]. The purpose of this paper is to describe the first case of collagenous gastritis occurring in a Tunisian male who presented with severe anemia.

## Patient and observation

**Figure 1:**
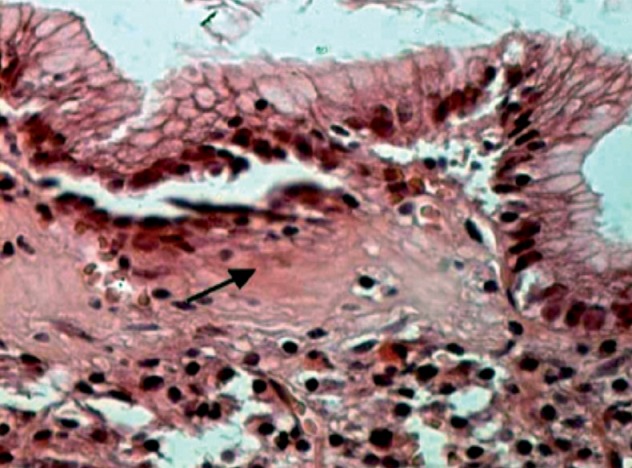
HE stain x 400: Thick collagenous subepithelial band. Detachment of the overlying epithelium. Inflammatory infiltrate in the lamina propria

**Figure 2:**
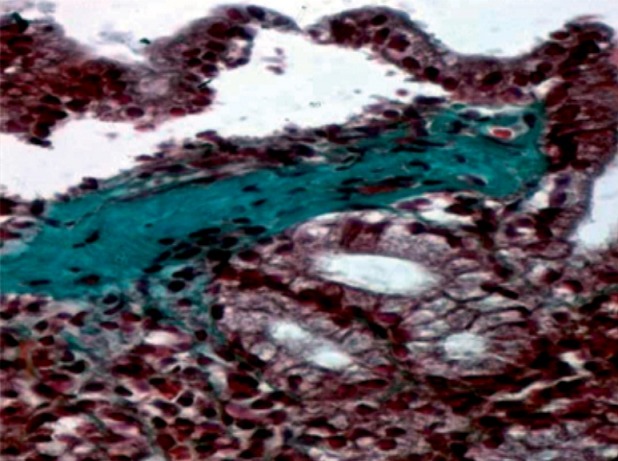
Trichrome stain: thick subepithelial collagenous band

A 19-year-old man presented in the department of Internal Medicine in July 2008 with dizziness and gradually increasing abdominal pain. He had had an intermittent abdominal pain without diarrhea or gastrointestinal bleeding for one year prior to the admission. The patient had no medical or surgical history. Physical examination revealed conjunctival pallor. Apart from this, no abnormalities was detected, especially no abnormality on vital signs, no hepatosplenomegaly or lymphadenopathy. Laboratory tests revealed hemoglobin of 6g/dL, a hematocrit of 20%, a mean corpuscular volume of 64 fl, a total leukocyte count of 4000/μL and a platelet count of 252 10³/μL. He had iron deficiency with ferritin of 1, 3 ng/mL. Folic acid and vitamin B12 levels were normal. Protein electrophoresis showed a total protein of 5.3g/dL and serum albumin of 2.5g/dL. The lactate dehydrogenase level was 743 U/L. Antigliadin, anti-endomysial and tissue transglutaminase antibodies were negatives. Total Immunoglobulin A level was normal. Upper gastrointestinal endoscopy revealed a nodular and congestive gastric mucosa. Ileocolonoscopic examination was normal. Biopsy specimens of mucosa taken from stomach showed a thick subepithelial collagenous band with an inflammatory infiltrate in the lamina propria including lymphocytes and plasma cells. The overlying epithelium was focally detached (Figure 1). A trichrome stain confirmed the collagenous nature of the band (Figure 2). These features are consistent with the diagnosis of collagenous gastritis. Biopsy specimens obtained throughout the colon and ileum was normal. The patient was diagnosed with collagenous gastritis. He started on prednisone 40 mg per day in September 2008. Two months later, he had significant clinical and biological improvement with disappearance of the abdominal pain and an increase on hemoglobin level at 10g/dL. A progressive decrease of prednisone was entertained after 2 months of full dose (40 mg per day) with a maintained clinical remission. However, a persistence of the collagenous gastritis was noted on the biopsy specimens taken from the gastric body and the antrum and realized 5 months after the treatment. The last check of the patient was in October 2010, after 18 months of stopping prednisone. The body mass index was stable (21 kg/m²) and the hemoglobin level rose to 14 g/dL. A third upper gastrointestinal endoscopy with biopsy was performed, the findings remained unchanged.

## Discussion

Collagenous gastritis is microscopically defined by the presence of a subepithelial collagenous band greater than 10 μm in thickness associated to entrapping dilated capillaries and inflammatory infiltration within the lamina propria [7]. Nielsen and al [8], distinguished two subtypes of proximal collagenous according to anatomical localization and symptoms. The first one is collagenous gastritis: it affects children and young adults and it is characterized by gastric involvement in the form of predominant nodular gastritis. The second one is collagenous sprue: it affects adults with lesion in the proximal small bowel. Collagenous colitis can be observed in both subtypes, but it is more common in the adult form. The symptoms of the adult form are diarrhea and weight loss from malabsorption [8]. The first subset is different from adult cases by the severity of the presentation including anemia and severe iron deficiency [6, 9, 10]. This anemia is probably due to gastric bleeding that may be caused by dilated capillaries entrapped in the subepithelial fibrous bands [9]. The clinicopathologic and endoscopic features of our patient are like pediatric cases of CG reported in the literature [7, 8, 11]. The etiology and the pathogenesis of this disorder still completely unknown [12, 13]. An immune mediated process has been suggested [14] as a mechanism because of coexisting immune related disorders in several patients such as collagenous colitis, lymphocytic colitis and celiac disease and constant signs of immune activation in gastric biopsy from patient with CG [7, 10, 15]. On the other hand, the presence of intraepithelial lymphocytic infiltrate and over expression of human leukocyte antigen DR and CD25 suggest immune processes [10]. In fact, in our patient, gastric biopsies showed signs of immune activation represented by the presence of cells expressing CD25 in the lamina propria and an increased number of intraepithelial T lymphocytes (expressing CD3). The collagen deposition may result from activated immune cells producing cytokines and growth factors, stimulating the production or reducing the turnover of extracellular matrix [7]. There is no established standard therapy for CG because of its unknown etiopathogenesis. Many treatment modalities such as PPIs, steroids, mesalazine, azathioprine have been tested [2]. In the pediatric form, both corticoids and PPIs are presently the drugs of choice [6]. In our case, the treatment with prednisone 40 mg/d for 6 weeks with subsequent tapering and cessation led to rapid clinical remission which still sustained after 18 months. But the histological lesions remain unchanged. In cases reporting long term follow up, the collagen deposits still unchanged or become thicker secondary to continued inflammation, also there was no evidence of the transformation of the pediatric form to adult form among these case reports [2].

## Conclusion

Collagenous gastritis is a rare disorder. Two subsets are individualized depending on the age of patient. Our Patient presented the subset occurring in children and young adults. The origin of the disease remains unknown. Lesions may be the result of a local immune process. Specific therapy has not been established, glucocorticosteroid may be helpful to relieve symptoms in collagenous gastritis patients.

## Competing interests

The authors declare no competing interest.
